# Colonization with *Escherichia coli* EC 25 protects neonatal rats from necrotizing enterocolitis

**DOI:** 10.1371/journal.pone.0188211

**Published:** 2017-11-30

**Authors:** Debi M. Thomas, Brandon Bell, Stephanie Papillon, Patrick Delaplain, Joanna Lim, Jamie Golden, Jordan Bowling, Jin Wang, Larry Wang, Anatoly V. Grishin, Henri R. Ford

**Affiliations:** 1 Division of Pediatric Surgery, Children’s Hospital Los Angeles, Los Angeles, California, United States of America; 2 Department of Surgery, University of Southern California, Los Angeles, California, United States of America; 3 Division of Pathology, Children’s Hospital Los Angeles, Los Angeles, California, United States of America; Gaziosmanpasa University, TURKEY

## Abstract

Necrotizing enterocolitis (NEC) is a significant cause of morbidity and mortality in premature infants; yet its pathogenesis remains poorly understood. To evaluate the role of intestinal bacteria in protection against NEC, we assessed the ability of naturally occurring intestinal colonizer *E*. *coli* EC25 to influence composition of intestinal microbiota and NEC pathology in the neonatal rat model. Experimental NEC was induced in neonatal rats by formula feeding/hypoxia, and graded histologically. Bacterial populations were characterized by plating on blood agar, scoring colony classes, and identifying each class by sequencing 16S rDNA. Binding of bacteria to, and induction of apoptosis in IEC-6 enterocytes were examined by plating on blood agar and fluorescent staining for fragmented DNA. *E*. *coli* EC 25, which was originally isolated from healthy rats, efficiently colonized the intestine and protected from NEC following introduction to newborn rats with formula at 10^6^ or 10^8^ cfu. Protection did not depend significantly on EC25 inoculum size or load in the intestine, but positively correlated with the fraction of EC25 in the microbiome. Introduction of EC25 did not prevent colonization with other bacteria and did not significantly alter bacterial diversity. EC25 neither induced cultured enterocyte apoptosis, nor protected from apoptosis induced by an enteropathogenic strain of *Cronobacter muytjensii*. Our results show that *E*. *coli* EC25 is a commensal strain that efficiently colonizes the neonatal intestine and protects from NEC.

## Introduction

Necrotizing enterocolitis (NEC) is a devastating inflammatory intestinal disease primarily affecting premature infants, with risk inversely related to birth weight and gestational age [1]. With an incidence of 2–5% in low-birth weight neonates, it is associated with serious morbidity and mortality [2]. Despite advances in neonatal care and surgical intervention, about 30% of affected neonates will die [3]. In cases of pan-necrosis, the rate of death is 100% [4]. Mainstays of treatment currently center on medical management such as bowel rest, fluid resuscitation, parenteral nutrition, and broad-spectrum antibiotics. Severe NEC (Bell Stage ≥ II) may require surgical intervention and has worse outcomes including intestinal strictures, short bowel syndrome, stoma complications, poor growth, and neurodevelopmental challenges [5].

The pathogenesis of NEC remains poorly understood, but in the setting of low birth weight and prematurity, significant risk factors include an immature gut barrier, absent or poorly functioning immune defenses, formula feeding, microbial dysbiosis, and hypoxic/ischemic conditions. There has been increasing interest in the role of intestinal microbiota in NEC [6]. Studies in gnotobiotic animals revealed that bacteria play a key role in the pathogenesis of NEC [7–9]. Although no single pathogen has been implicated as a causative agent of NEC [10], strains of *Helicobacter* [11], *Clostridium* [12] and *Cronobacter* [13] increased NEC pathology upon introduction to laboratory animals. Lack of knowledge about specific NEC pathogens has led to the prolonged use of empiric antibiotics, which by itself may be conducive to NEC [14]. Taken together, these findings strongly implicate bacteria in the pathogenesis of NEC.

Culture-based and culture-independent methods have been used to evaluate changes in microbiota in clinical and experimental NEC [15]. However, no characteristic or pattern of perinatal colonization can be reliably attributed to the disease [10].

While some bacteria may act as NEC pathogens, others may be either protective or innocuous. Protective properties of bacteria belonging to the genera *Lactobacillus* and *Bifidobacterium* have been extensively researched. While some trials demonstrated beneficial effects of these bacteria in NEC [16], others found no effect [17] or even adverse effects [18]. Because species of probiotic bacteria, formulations, and dosages were different in different studies, data from these studies often cannot be compared [19]. Little is known about effects of probiotics on the intestinal microbiota or mechanisms of protection. Due to these uncertainties, there is no consensus at this time about the use of *Lactobacillus* or *Bifidobacterium* for prophylaxis or treatment of NEC [20, 21]. Even less is known about what constitutes “normal” vs. “abnormal” gut colonization, or how the microbiota can be manipulated to prevent NEC.

We hypothesize that various bacteria colonizing the immature gut may act as either NEC promoting opportunistic pathogens or NEC-abating probiotics. The goal of this study was to evaluate the ability of a naturally occurring early colonizer, *E*. *coli* EC25, to influence intestinal microbiota and pathology in the rat model of NEC. EC25 efficiently colonized the neonatal gut and protected from NEC. Results of this study provide an insight into the role of early microbiota in NEC.

## Materials and methods

### Reagents and media

All common reagents were from Sigma-Aldrich (St. Louis, MO). Fetal bovine serum (FBS), *Hin*dIII restriction endonuclease, Luria-Bretani (LB), blood agar base, McConkey agar base, and thioglycollate medium were from Sigma-Aldrich. Defibrinated sheep blood was from Hemostat Laboratories (Dixon, CA). IκBα antibody was from Cell Signaling Technology (Danvers, MA). Gram staining kit was purchased from Becton Dickinson Biosciences (San Jose, CA). Dulbecco-modified Eagle Medium (DMEM) was from Mediatech (Manassas, VA). *Cronobacter muytjensii* cat.# 51329 and IEC-6 cells were from ATCC (Manassas, VA).

### Rat NEC

**Fig 1:**
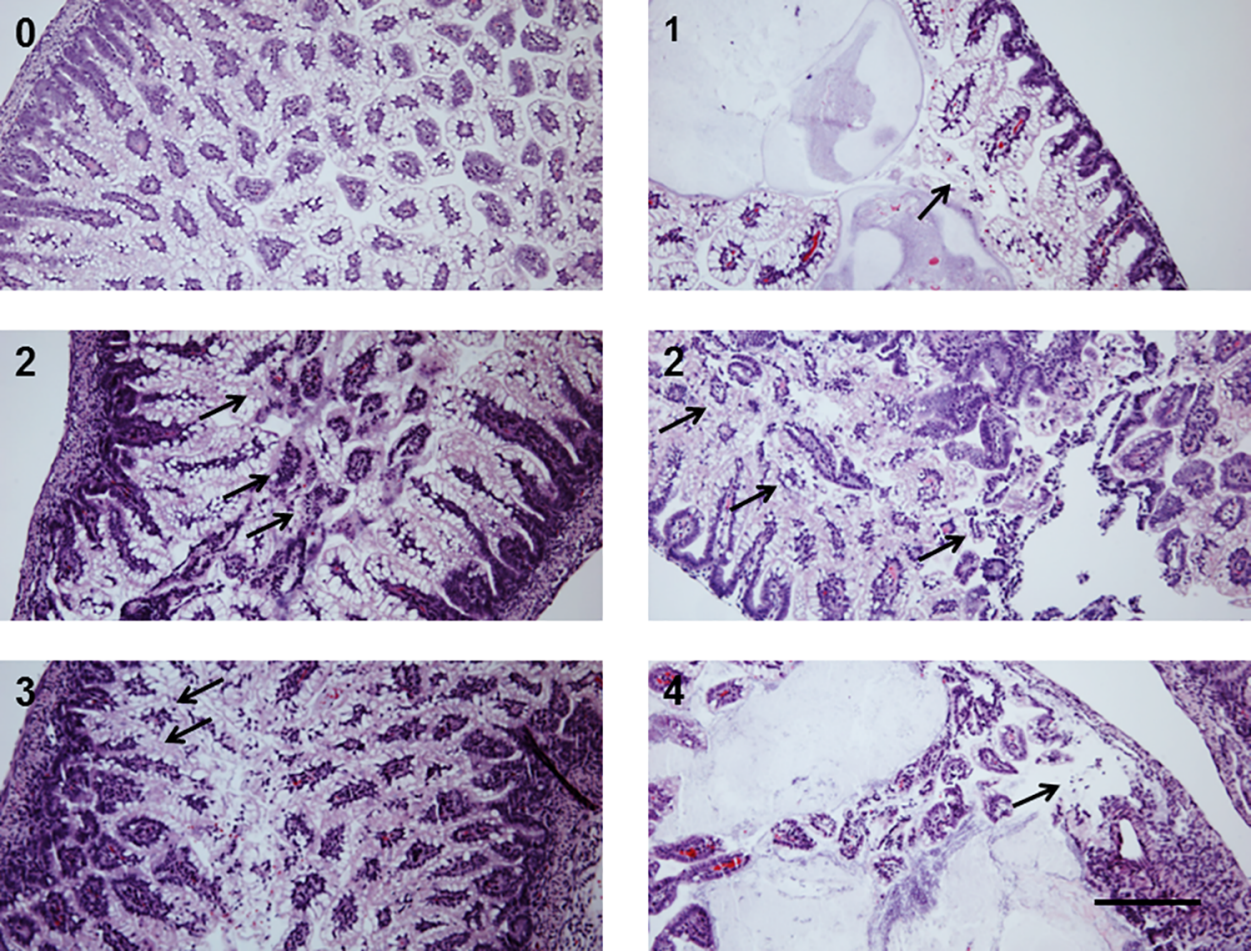
NEC pathology scores. Scores were assigned based on the extent of epithelial damage, as shown in the representative images. 0, normal epithelial architecture; 1, epithelial sloughing; 2, destruction involving distal half of the villi; 3, destruction beyond distal ½ of the villi; 4, complete obliteration of the epithelium. Arrows indicate damaged area. Bar = 100 μm.

All animal experiments were approved by the Institutional Animal Care and Use Committee (IACUC) of Children’s Hospital Los Angeles. Timed pregnant Sprague-Dawley rats were obtained from Charles River (Hollister, CA) or Harlan Laboratories (Livermore, CA) at E15 and allowed to deliver naturally. At the time of delivery, the neonates were randomized into control and treatment groups, and placed in a 30°C and 90% relative humidity incubator. NEC was induced by formula feeding and hypoxia (FFH, 5% O_2_, 95% N_2_ for 10 min) as previously described [22]. Formula was prepared under sterile conditions and tested for bacterial contamination. Measures were taken to minimize introduction of extraneous bacteria during handling and feeding. Bacteria were added to formula from a fresh overnight culture grown in LB medium. Bacteria-spiked formula was stored on ice for no longer than 20 h. Animals were euthanized on day of life 4 by decapitation following pentobarbital anesthesia (pups) or by CO_2_ (adults). A segment of the distal ileum was fixed in 4% buffered formalin. 5-micron hematoxylin-eosine-stained sections were scored by a pathologist blinded to groups as follows: normal architecture, 0; epithelial sloughing with or without sub-mucosal edema or neutrophil infiltration with villi intact, 1; destruction involving upper halves of the villi, 2; destruction involving lower halves of the villi with crypts intact, 3; complete disruption of epithelial architecture with or without intestinal wall perforation, 4 (Fig 1). The damage was scored in the worst affected area. Scores of 2 or more were considered positive for NEC.

### Quantification and identification of intestinal bacteria

Freshly excised 1 cm samples of small intestine (~25 μl volume) were homogenized on ice in 1 ml of sterile 0.9% NaCl, and serial dilutions of the homogenate were immediately plated on 6% sheep blood agar. Following 2 day incubation at 37°C, bacterial colonies were classified under 4 x magnifications according to their appearance, and numbers in each class were counted. Typically, colony classes of characteristic shape, size, color, and type of hemolysis were readily distinguishable after two days of growth. In the case of doubt, similarly looking colonies were re-streaked side-by-side, and, if the emerging colonies looked the same, they were considered the same strain. Pure cultures established by repeated re-streaking of well-separated colonies on blood agar plates were frozen at -70°C. Strains of the same species were distinguished by colony appearance and, if necessary, by comparison of DNA restriction fragment patterns. Only classes constituting 0.5% or more of the total population were scored. Bacterial loads (cfu/ml) in tissue homogenates were calculated based on the dilution factor. To identify bacteria, the V6-V8 segment of the 16S rRNA gene was PCR-amplified using the primers AGAGTTTGATCCTGGCTCAG and ACGGCTACCTTGTTACGACTT, and sequenced. Quality-evaluated sequences were queried against the non-redundant nucleotide (nt) database using the NCBI BLAST algorithm. Whenever species identification was ambiguous, Gram staining followed by group-specific biochemical and physiological tests [23] were used for disambiguation. Shannon’s diversity index (SDI),

$$H^{'}=-\sum_{i=1}^{R}p_{i}\ln p_{i}$$

where *p*_*i*_ is proportion of species *I* in the population, and *R* is number of different species, was calculated online at EasyCalculation.com.

### Restriction endonuclease patterns of bacterial DNA

Bacteria grown on blood agar were re-suspended in DNA buffer (10 mM Tris pH7.5, 100 mM NaCl, 1 mM EDTA) and homogenized by vortexing at full speed with 200 micron glass beads for 5 min. After addition of sodium dodecyl sulfate and proteinase K to 1% and 20 μg/ml, respectively, samples were incubated at 50°C for 8–12 h, followed by 80°C for 5 min. After sequential deproteinization with equilibrated phenol pH8.0 and chloroform, nucleic acids were precipitated with 2 volumes of cold ethanol, washed with 70% ethanol, and dissolved in ddH_2_0. 5 micrograms of bacterial DNA were digested with 10 units of the *Hin*dIII restriction endonuclease and 20 μg/ml pancreatic RNAse. Digestion products were resolved on 0.7% agarose Tris-acetate ethidium bromide gels. Gels were illuminated with UV and photographed using GelDoc GS700 (Bio-Rad, Hercules, CA).

### Bacterial adhesion and apoptosis tests in cultured enterocytes

IEC-6 enterocytes, passages 20–30, were grown in DMEM supplemented with 5% FBS at 37°C in a humidified air containing 5% CO_2_, to 70–90% confluence. Bacteria grown overnight to saturation in LB were diluted in DMEM and added to IEC-6 cells. Bacteria-containing medium was replaced every 2 h to prevent bacterial overgrowth. For bacterial binding, IEC-6 cells were washed 3 times with cold DMEM, collected, and homogenized in a Dounce homogenizer. Serially diluted homogenates were plated onto LB agar for bacterial colony counts. Binding was expressed as fraction of bound bacteria. For apoptosis assay, cells were fixed with formalin and stained using the In Situ Cell Death Detection kit as recommended by the manufacturer (Roche Diagnostics, Indianapolis, IN). Following staining, cells were mounted in medium containing diamidino phenyl indole and examined by fluorescence microscopy. Levels of apoptosis were expressed as percentage of apoptotic nuclei.

### Western blotting

Total protein was extracted from cultured cells by incubation with lysis buffer (20 mM Tris pH 7.5, 0.1 M NaCl, 1% NP-40, 0.5% sodium deoxycholate, 0.1% sodium dodecyl sulfate (SDS), 1 mM phenylmethylsulfonyl fluoride) at 0°C for 10 min, followed by 10 min centrifugation at 10,000xg. 50 μg protein samples were resolved by electrophoresis through 10% SDS-polyacrylamide gels. Protein transfer, membrane blocking, antibody incubation, and film imaging were performed as recommended by antibody manufacturer.

### Statistical analysis

All analyses were performed using GraphPad Prism 6 (GraphPad Software, Inc., La Jolla, CA). Student’s *t* test and χ^2^ test were used for pairwise comparisons of parametric and non-parametric data. Differences were considered significant at p<0.05.

## Results

### NEC is not associated with characteristic patterns of microbiota

In attempt to establish connection between microbiota and NEC, we examined histopathology and intestinal bacteria in 150 neonatal Sprague-Dawley rats from 17 litters, breast-fed (36) or subjected to the NEC-inducing regimen of formula feeding and hypoxia (FFH, 114). Mothers of these neonates were sourced from two different suppliers to ensure diversity of microbiota. Because we intended to examine effects of newly identified bacterial strains in experimental NEC, plating on blood agar was used both to enumerate bacteria and to isolate pure cultures. Previous studies of early postnatal microbiota reported that culture-dependent and culture-independent methods yield similar results[24,25].

**Fig 2:**
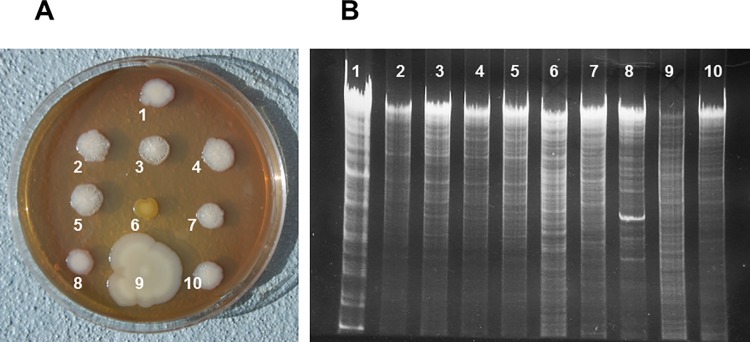
NEC scores and numbers of identified strains. A Distribution of NEC scores in Charles River (solid bars) and Harlan Labs (open bars) rats. B, Numbers of different bacterial strains found in litters. C, Numbers of bacterial strains found in individual animals. B and C show data for combined breast fed and formula fed population. D, average logarithms of total bacterial loads in animals with NEC (scores 2–4, n = 60) and without NEC (scores 0–1, n = 72).

The overall incidence of NEC in the FFH group was 44/114, or 39%. Animals from Harlan Labs had significantly higher incidence and scores of NEC than those from Charles River (Fig 2A) (34/53 vs. 10/61, p = 0.002, χ^2^ test). The number of bacterial strains detected varied from 1 to 21 in litters and from 0 to 9 in individual animals, with median values of 8 and 1, respectively (Fig 2B and 2C, S1 Data File). Animals carrying only one identifiable bacterial strain were the predominant group (57/150). Each litter had its unique set of bacterial strains, except two litters of Charles River rats that harbored the same strain of *E*. *coli*. There were no bacterial strains shared by Charles River and Harlan rats. Total bacterial loads varied from undetectable to 10^11^ cfu/ml; the average logarithms of bacterial loads after four days of formula feeding and hypoxia did not differ significantly between animals with or without NEC (3.5±0.47 vs. 3.8±0.51; Fig 2D; S1 Data File). Most of the bacterial species identified were likely mammalian commensals, with a few environmental saprophytes (Table 1).

**Table 1:** Bacterial species and strains found in 4 day old rats.

Species	Commensal	Pathogen	Environmental	Strains
*E*. *coli*	+	+		14
*Escherichia* sp.	+	+		1
*E*. *fergusonii*	+	+		1
*Shigella* sp.	+	+^1^		3
*S*. *flexneri*	+	+		1
*Stenotrophomonas maltophila*		+	+	1
*Cronobacter sakazakii*		+	+^2^	2
*Proteus mirabilis*		+	+	1
*Enterococcus faecalis*	+	+		5
*E*. *faecium*	+	+		1
*E*. *avium*	+	+		1
*E*. *hirae*	+	+		1
Micrococcus luteus			+	1
*Micrococcus* sp.		+	+	1
*Stapylococcus aureus*	+	+		3
*Staphylococcus* sp.	+			4
*S*. *epidermidis*	+	+		1
*S*. *haemolyticus*	+	+		2
*S*. *lugdunensis*	+	+		1
*S*. *carnosus*	+		+^2^	1
*S*. *cohnii*	+			2
*Bacillus megatherium*			+	2
*Bacillus* sp.			+	2
*B*. *pseudofirmus*			+	1
*B*. *aryabbattai*			+	1
*B*. *amyloliquefaciens*			+	1
*Lactobacillus ruminis*	+			1
*Neisseria lactamica*	+			1
Unidentified				24

^1^Pathogenic only in primates

^2^Food-associated bacteria

**Fig 3:**
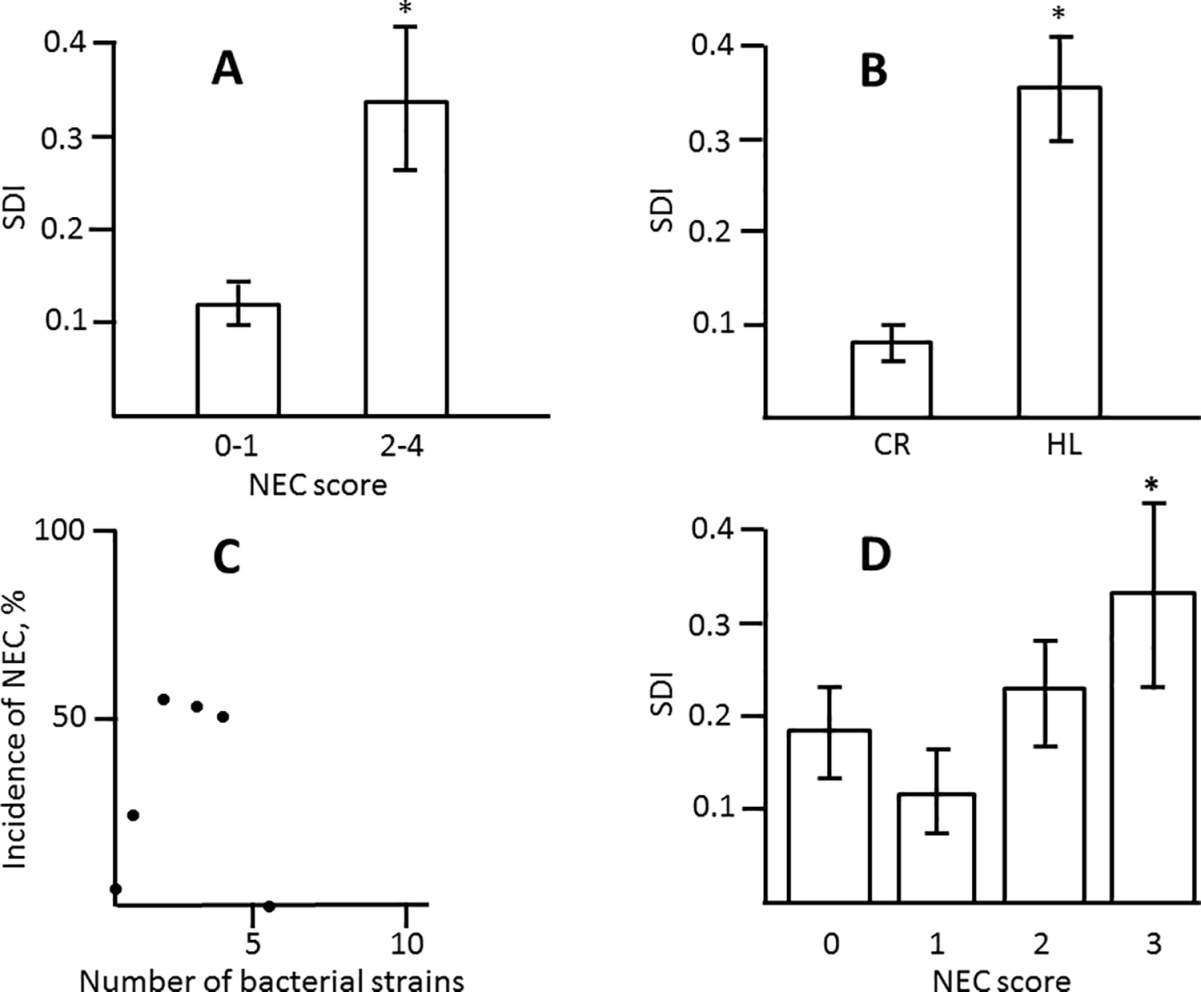
Bacterial diversity in study cohorts. A, Shannon’s diversity indices of bacterial populations in Charles River (CR, n = 58) and Harlan Labs (HL, n = 74) 4 day old rats. B, Incidence of NEC (score ≥2) in formula fed rats harboring different numbers of bacterial strains.

Previous studies reported association of NEC with decreased bacterial diversity [26–28] or prevalence of *Enterobacteriaceae* [27–30]. Harlan Labs animals harbored more diverse bacteria than Charles River animals (Fig 3A). However, within Charles River and Harlan Labs sub-populations, there were no significant differences in Shannon’s diversity indices between animals with NEC and with no NEC (p>0.8, Sudent’s *t* test). There was no apparent correlation between NEC incidence and number of bacterial strains in litters (Fig 3B). In the FFH group, the *Enterobacteriaceae* were predominant colonizers in 64% (27/42) of healthy animals, but only in 32% (11/34) of animals with NEC. Thus, we did not find association of NEC with total bacterial loads, microbiota diversity, or colonization with *Enterobacteriaceae*.

### Colonization with *E*. *coli* EC 25 was not associated with NEC

Data from hospital outbreaks and our studies in the rat model of NEC indicated that some of the strains of *Cronobacter muytjensii* or *C*. *sakazakii* may act as NEC pathogens [13, 31]. Based on these observations and published data [32], we hypothesized that a variety of opportunistic pathogens may increase the risk of NEC upon colonization of a susceptible host. By contrast, colonization with innocuous microbes should decrease NEC via competition with pathogens. To identify potentially beneficial colonizers, we looked for the strains of bacteria predominately found in healthy neonatal rats after four days of formula feeding–hypoxia. In one of the litters of Charles River rats, all the animals were healthy, and all of them carried only one bacterial strain, the isolate 25 (S1 Data File). By querying of 16S rRNA, this strain was found to be closely related to a number of *Escherichia*, *Shigella*, and *Citrobacter* strains. Loads of the strain 25 varied in the littermates from 4.3x10^7^ to 7.8x10^10^. Strain 25 was also found in breast fed Charles River pups from a different litter, but not in NEC pups (S1 Data File). Since even relatively high loads of the strain 25 did not lead to NEC, we reasoned that it is a good candidate for a non-pathogenic colonizing bacterium.

To definitively classify strain 25, a battery of 24 bacteriological tests was employed (S1 Table). Since results of all the tests were consistent with the strain 25 being *E*. *coli*, we designated it as EC25.

**Fig 4:**
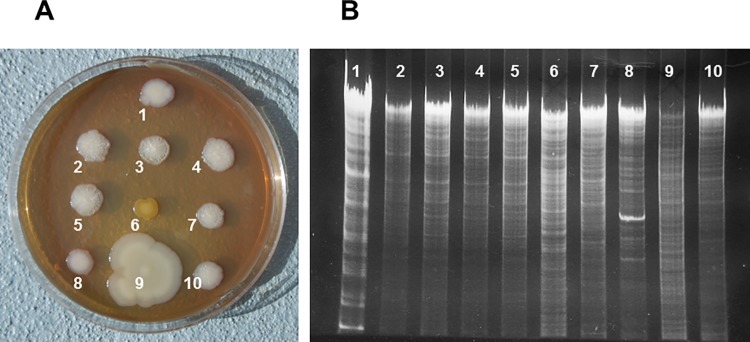
Colony morphology and DNA restriction fragments of bacterial strains. A, Colony morphology. EC25 colonies (2–5) have characteristic strong wrinkles and small central button. Other *E*. *coli* strains have smooth (1, 8), wrinkled with large central button (7), or weakly wrinkled (10) colonies. Colonies of other species (6, 9) are grossly different from those of *E*.*coli*. B, Electropherograms of *Hin*dIII-digested DNA. 1, *E*. *coli* Di114; 2–5, *E*. *coli* EC25; 6, *Micrococcus luteus*; 7, *E*. *coli* FUA1242; 8, *E*. *coli* DSM1103; 9, *Citrobacter freundii*; 10, *E*. *coli* NRG85C.

EC25 had characteristic colony morphology and DNA restriction pattern, which allowed distinguishing it from other strains of *E*. *coli* and other bacterial species. Upon prolonged incubation (5 days), colonies of EC25 developed characteristic strongly wrinkled appearance with small central button (Fig 4A, 2–5). EC25 had a unique pattern of *Hin*dIII restriction fragments (Fig 4B, 2–5).

### EC 25 decreases the incidence and severity of NEC

**Fig 5:**
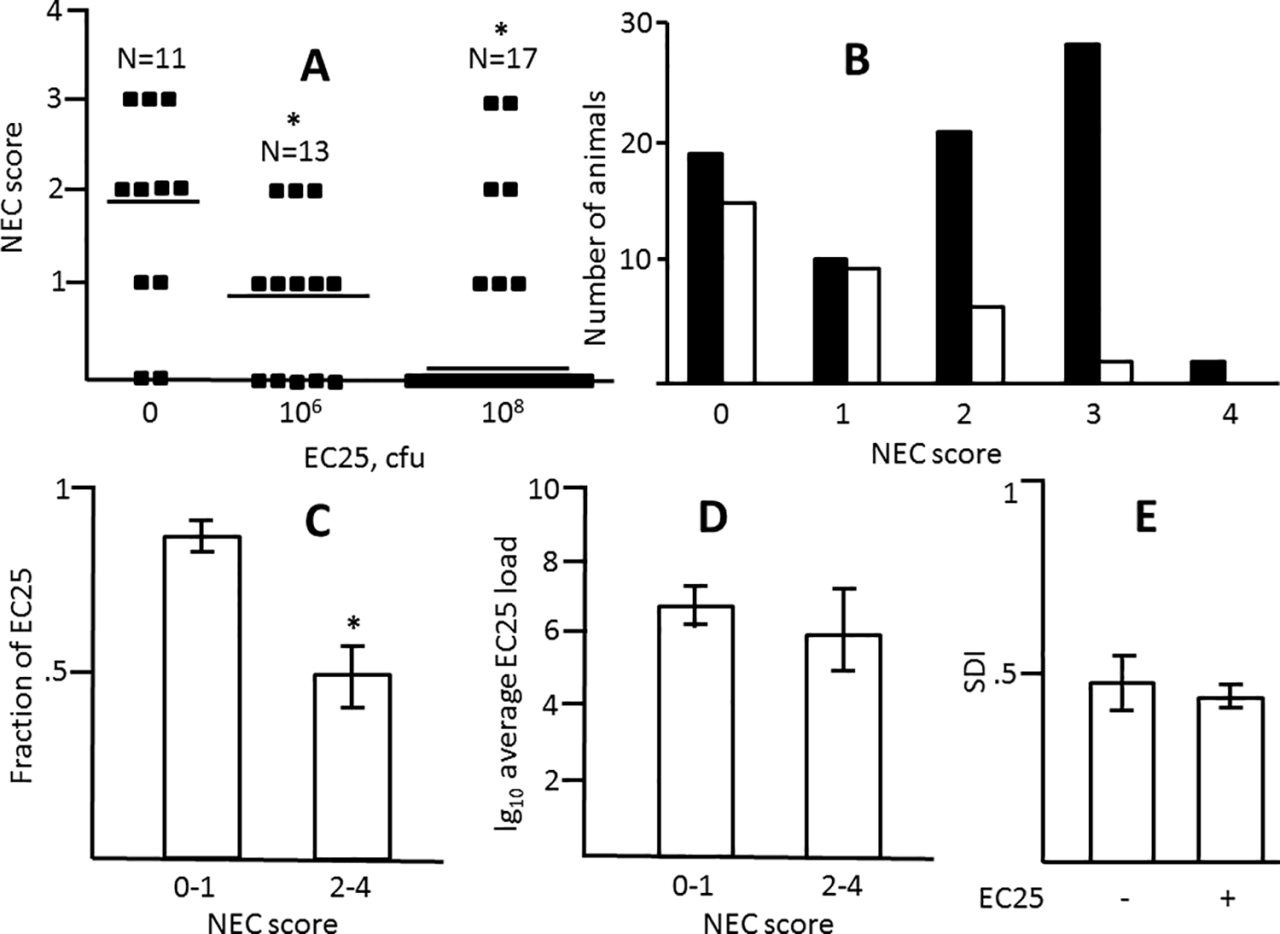
Effects of *E*. *coli* EC25 on NEC pathology. A, Distribution of NEC scores in Harlan Labs rats that received 0, 10^6^, and 10^8^ cfu of EC25 per feeding. Horizontal bars are medians. *, significant difference from the group that received no EC25 (p<0.05). B. NEC scores in control Harlan Labs rats (solid bars) and those that received EC25 (open bars). C, Average fractions of EC25 in microbimes of NEC (score 2–4, n = 8) and no NEC (score 0–1, n = 24) rats. All animals received 10^6^ or 10^8^ cfu of EC25. *, significant difference, p = 0.014. D, Average logarithms of total EC25 loads in no-NEC (n = 24) and NEC (n = 8) animals. E, Average SDI with (n = 32) and without (n = 80) supplementation with EC25.

To examine whether EC25 is capable of protecting from NEC, we modified our NEC-inducing regimen by adding this strain to formula so as to introduce 10^6^ or 10^8^ cfu per feeding. Harlan Lab rats were used in this experiment since they are more susceptible to NEC. At either concentration, EC25 significantly reduced NEC scores (Fig 5A and 5B; S1 Fig; S2 Data File). Although the median NEC score was lower at higher dose of EC25, the difference between scores at lower and higher concentrations of EC25 was not significant (Fig 5A). Of the animals that received EC25, those that developed NEC had significantly lower fraction of EC25 in their microbial communities compared to healthy animals (Fig 5C; S2 Data File). However, there were no significant differences in total loads of EC25 between healthy and sick animals (Fig 5D). Also, no significant differences were found in either total load or percentage of EC25 between groups that received different concentrations of EC25 (data not shown). Thus, EC25 protected neonatal rats from NEC, and, although this protection was not dose-dependent with the two doses tested, it tended to occur when this strain established itself as the major constituent of the gut microbiota.

### EC25 is capable of colonizing the neonatal intestine

Next, we quantitatively characterized the ability of EC25 to colonize the intestine. EC25 was recovered from the majority (29 out of 30) of neonates who received these bacteria with formula. Moreover, EC25 became the predominant colonizer in 25 out of 30 animals. The titers of EC25 in the intestines of these animals after 4 days of feeding with bacteria-spiked formula varied from 10^3^ to 10^12^ cfu/ml. Introduction of EC25 did not prevent colonization with other bacteria in 23 out of 29 animals and did not significantly alter bacterial species diversity (Fig 5E; S2 Data File). Thus, EC25 is capable of colonizing the neonatal intestine when introduced with formula, but colonization with this strain does not prevent naturally occurring colonization with other bacteria.

### EC25 does not induce enterocyte apoptosis in vitro

**Fig 6:**
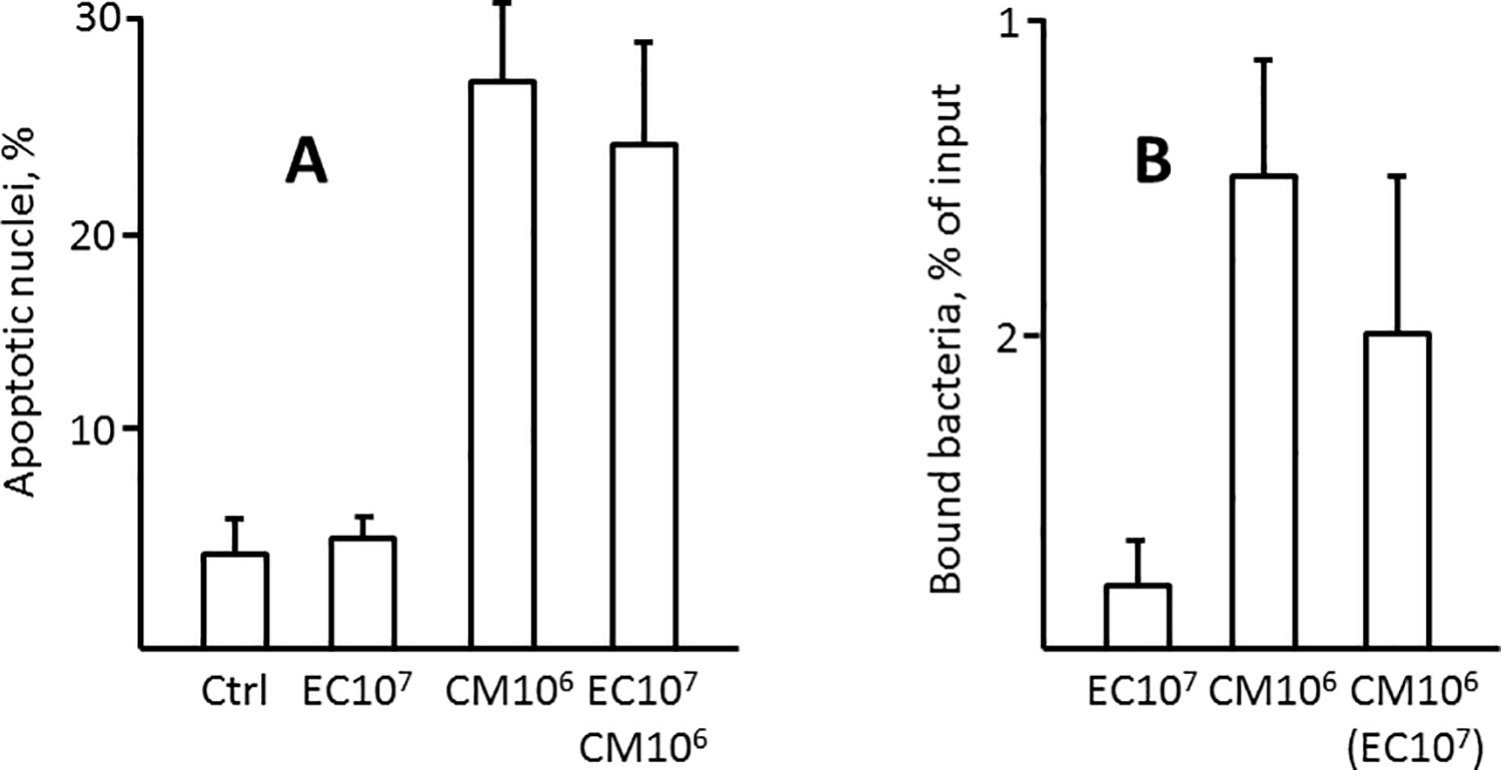
Induction of enterocyte apoptosis and enterocyte binding by EC25. A, Average levels of apoptosis in untreated IEC-6 cells, and cells treated with 10^7^ cfu/ml EC25, 10^6^ cfu/ml *C*. *muytjensii*, or combined EC25 and *C*. *muytjensii* for 6 h. 500 cells were scored in each treatment group. B, Average binding of EC25 and *C*. *muytjensii* to IEC-6 cells at the indicated bacterial inputs. In combined treatment, only binding of *C*. *muytjensii* was scored. All data are from at least 3 independent experiments.

*C*. *muytjensii* 51329, a well-characterized NEC pathogen, exhibits considerable cytotoxicity towards enterocytes in vitro [13]. Since EC25 protects from NEC, it is not expected to be cytotoxic. To test the cytotoxic properties of EC25, we examined its ability to induce apoptosis in IEC-6 enterocytes. Following incubation with EC25 under conditions that prevent bacterial overgrowth and depletion of growth medium, the levels of IEC-6 cell apoptosis did not increase significantly over medium-only control. By contrast, treatment with *C*. *muytjensii* 51329 under similar conditions induced massive enterocyte apoptosis. 10-fold excess of EC25 did not significantly reduce the induction of enterocyte apoptosis by *C*. *muytjensii* 51329 (Fig 6A, S2 Fig). Since cytotoxicity of *C*. *muytjensii* 51329 may be associated with its ability to adhere to enterocytes [13, 31], we asked whether EC25 could compete with *C*. *muytjensii* for binding. EC25 bound to IEC-6 cells at lower efficiency compared to *C*. *muytjensii* 51329, and did not significantly compete with the latter for binding (Fig 6B). These results indicate that EC25 is neither efficiently adherent nor cytotoxic to enterocytes, and it is not capable of reducing binding or cytotoxicity of the enteropathogenic bacterium.

We could not ascertain whether EC25 reduced NEC-associated epithelial apoptosis in vivo because apoptosis, like epithelial injury, is focal in this disease and therefore difficult to quantify. Generally, apoptosis tended to correlate with NEC score (S3 Fig).

Since innate immune response to the nascent microbiota, particularly to Gram-negative bacteria, could potentially contribute to NEC, we compared EC25 and other *E*. *coli* strains (K12 and several strains identified in this study) with regard to their ability to activate NF-κB, the key transcription factor in the innate immune response, in cultured enterocytes. EC25 activated NF-κB as efficiently as other *E*. *coli* strains, judged by degradation of the inhibitory subunit IκBα (S4 Fig). Thus, protection by EC25 is not due to decreased ability of this strain to induce innate immune response in enterocytes.

## Discussion

We have isolated and characterized the *E*. *coli* strain EC25 that was present as a naturally occurring colonizer in healthy neonatal rats following the NEC-inducing regimen of formula feeding and hypoxia. Since even high titers of EC25 were not associated with NEC, we hypothesized that this strain has protective properties. This hypothesis was tested by assessing the ability of EC25 to prevent NEC. When introduced with formula, EC25 efficiently colonized the small intestine of neonatal rats and reduced incidence and severity of NEC. This study unambiguously proves that EC25 is not a NEC pathogen: the strain was isolated from healthy animals, a pure culture was established, introduction of EC25 decreased NEC, and EC25 was re-isolated from the inoculated animals. *E*. *coli* EC25 is similar to the previously described *E*. *coli* Nissle 1917 in its ability to protect the intestine from infectious bacteria[33].

In addition to identifying of EC25 as an innocuous commensal, our study provides an insight into early microbiota and its connection to NEC. We have found that early bacterial populations are quite simple. One-strain populations were the predominant group, and no more than 9 strains were found in individual animals. The populations were highly diverse in species composition and bacterial loads. These findings are in agreement with previous studies [15]. NEC was not correlated with total bacterial loads. There was significantly higher bacterial strain diversity and paucity of *Enterobacteriaceae* in NEC animals compared to healthy ones, which is contrary to findings of several other reports [27–32]. The discrepancy is likely due to non-representative character of limited subject populations in this and other studies. Effect of bacterial diversity in NEC may depend on the context of specific bacterial populations. For example, if care is taken to reduce potentially pathogenic extraneous bacteria (e.g. SPF housing), low microbial diversity may not correlate with NEC. We believe that was the reason why we observed lower diversity population and lower incidence of NEC in rats from Charles River Laboratories, the facility that practices SPF standards. In fact, a number of studies did not find any correlation between NEC and *Enterobacteriaceae* or decreased bacterial diversity, or any other correlation between microbiota composition and NEC. Thus, like many others, we did not identify any microbiota pattern clearly associated with NEC.

Our experiments provide an insight into the earliest steps of bacterial colonization. From a microbiologist’s point of view, a neonate might be similar to a bioreactor providing more or less favorable environment for various bacteria, with those arriving first establishing themselves as first colonizers. Introduced shortly after birth, EC25 colonized the majority of neonates, and in this respect it behaved according to the bioreactor model. However, final titers of this strain in inoculated animals varied widely and did not depend much on the size of the inoculum. Although sometimes EC25 was able to establish itself as the sole colonizer, in most animals comparable titers of other bacteria were also present. Despite repeated introduction, EC25 failed to colonize the gut in two animals. These results indicate that bacterial colonization of the intestine is more complex than what is predicted by the bioreactor hypothesis.

Experiments in IEC-6 cells indicate that EC25 neither induces enterocyte apoptosis, nor it protects enterocytes from apoptosis caused by an enteropathogenic *C*. *muytjensii*. Binding of EC25 to enterocytes is relatively low. The ability of EC25 to protect from NEC did not significantly depend on its dose or intestinal load, but positively correlated with relative abundance of this strain in the intestinal microbiota (about 50% in NEC vs. 80% in the absence of NEC). Although some form of active protection, e.g. bacteriocins, protective peptides, or induction of host protective factors [34–37], cannot be excluded, our data is consistent with EC25 protecting from NEC passively, via competition with pathogenic bacteria for colonization of the intestine.

Microbiological analysis of the intestinal microbiota might have two limitations—low resolution and failure to identify unculturable bacteria. Due to the latter limitation there is no guarantee that some bacterial species of importance to NEC are not overlooked. However, the culture method is adequate for this particular study because early gut populations have low complexity, and because no unculturable species have been identified so far in these populations by culture-independent methods. Whenever culture-based and culture-independent methods were used in parallel to characterize early postnatal microbiota, they yielded similar results. Although culture methods are inferior in their throughput capacity to direct DNA sequencing methods, they have their advantages [38] and are indispensable whenever bacterial strains are to be isolated for examining their pathogenic properties.

Experimental design described here can be used to characterize other early colonizers. Most NEC microbiome studies do not go beyond identification of suspect pathogens or protective strains. As a result, these studies remain inconclusive. By introducing the suspect strains to neonatal rats and examining their effects on NEC, it is possible to make definite conclusions. Although experiments with bacterial cultures are time consuming, there is no alternative to them in the efforts to understand the role of bacteria in NEC.

## Supporting information

S1 Fig Typical hematoxylin-eosin-stained intestinal sections of 4 day old rats. (TIF)

S2 Fig Bacteria-induced apoptosis in IEC-6 cells. (TIF)

S3 Fig NEC-associated epithelial apoptosis. (TIF)

S4 Fig Degradation of IκBα following exposure of IEC-6 calls to different *E*. *coli* strains. (TIF)

S1 Table Results of EC25 bacteriological tests. (DOCX)

S1 Data File Bacteria found in 4 day old Charles River and Harlan rats. (XLS)

S2 Data File Effect of introduced EC25 on populations of intestinal bacteria. (XLSX)
