# Supplementary figures and images for: Colonization with Escherichia coli EC 25 protects neonatal rats from necrotizing enterocolitis
Source: PLoS One. 2017 Nov 30;12(11):e0188211. doi: 10.1371/journal.pone.0188211 (PMC5708813; doi:10.1371/journal.pone.0188211)

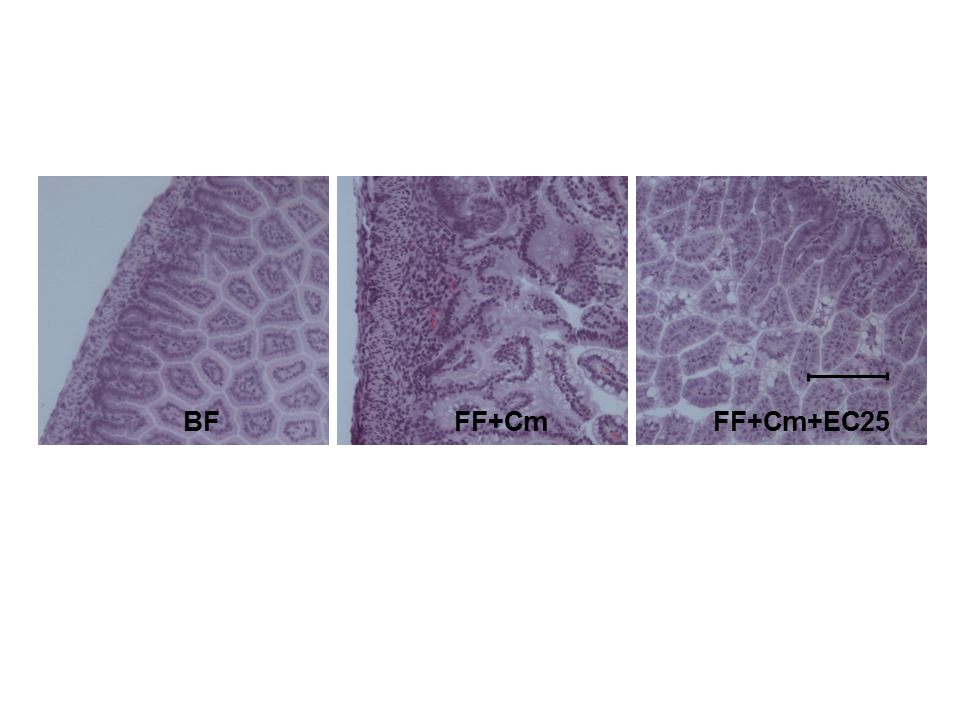

Supplement: S1 Fig — (TIF) [file pone.0188211.s001.tif]

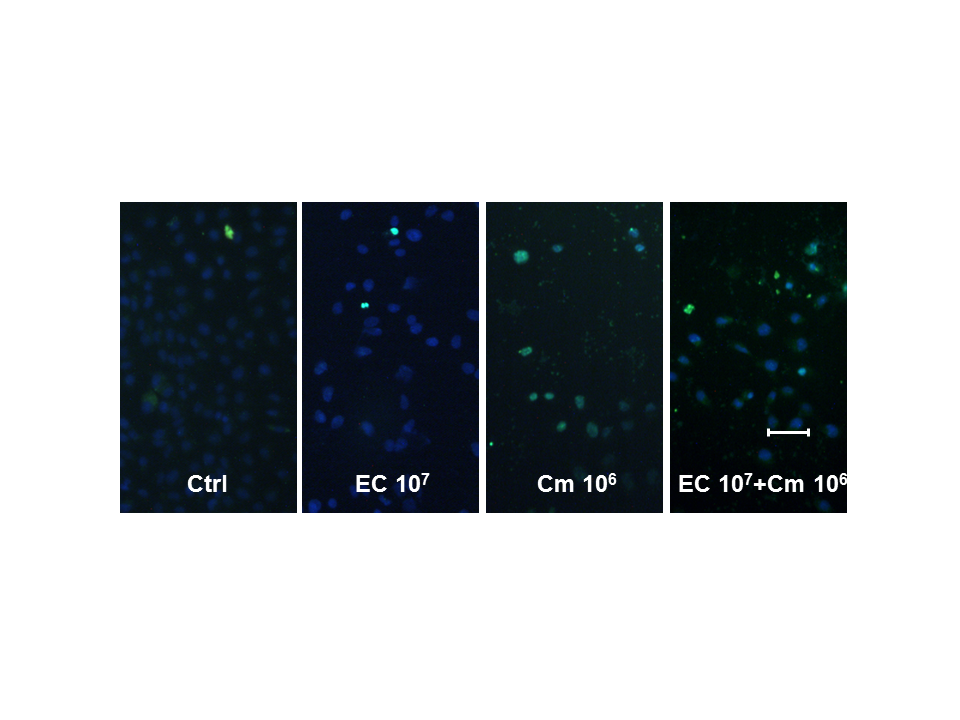

Supplement: S2 Fig — (TIF) [file pone.0188211.s002.tif]

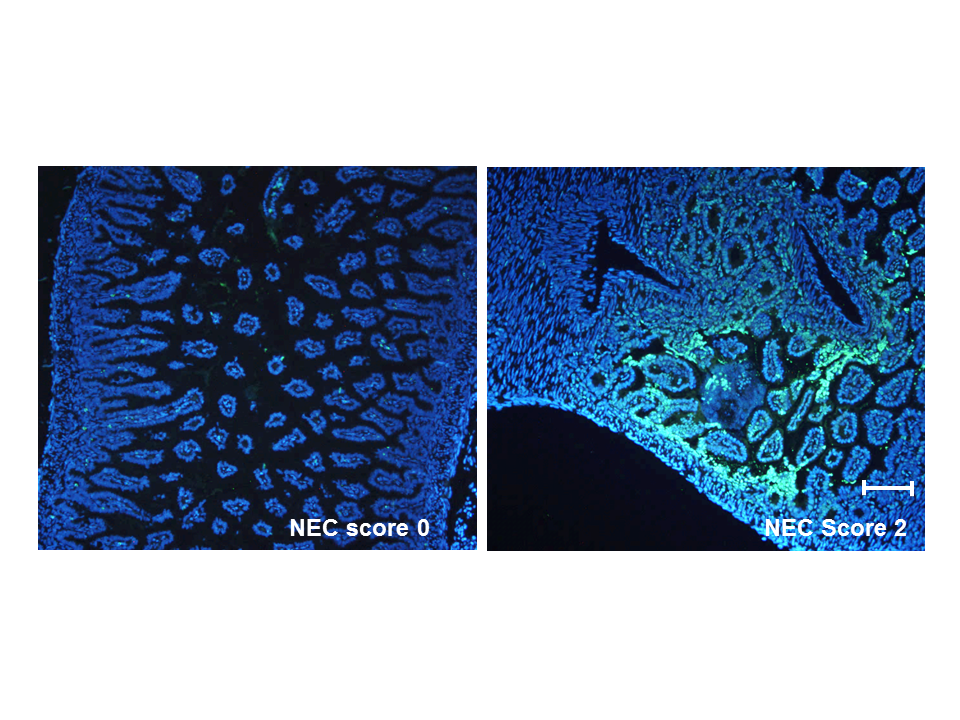

Supplement: S3 Fig — (TIF) [file pone.0188211.s003.tif]

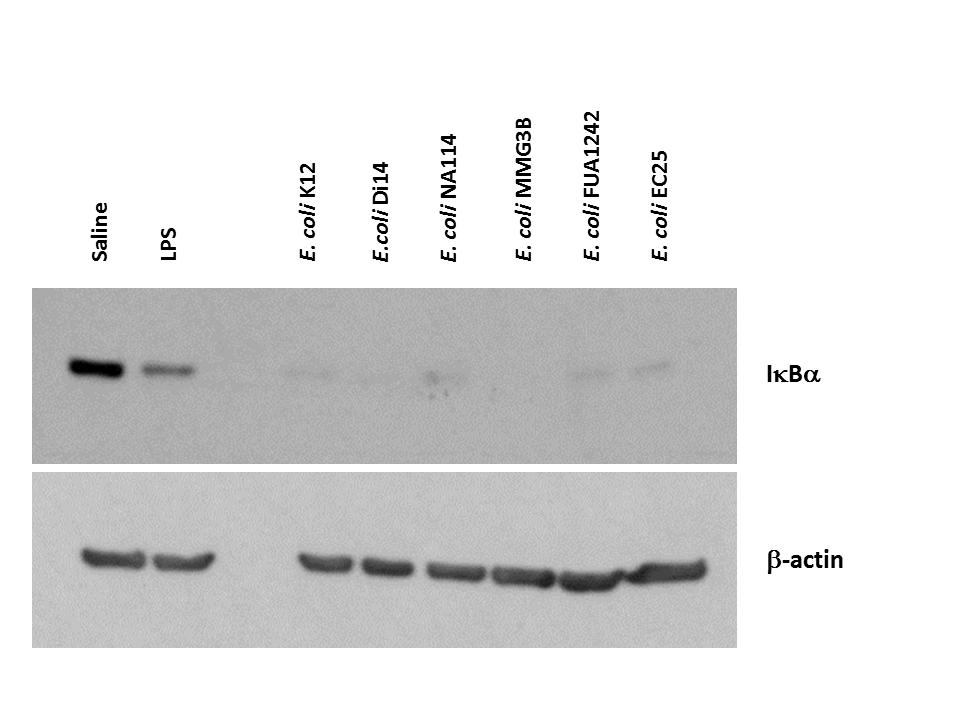

Supplement: S4 Fig — (TIF) [file pone.0188211.s004.tif]
