# Supplementary material for: Colonization with Escherichia coli EC 25 protects neonatal rats from necrotizing enterocolitis
Source: PLoS One. 2017 Nov 30;12(11):e0188211. doi: 10.1371/journal.pone.0188211 (PMC5708813; doi:10.1371/journal.pone.0188211)
Supplement: S1 Table — (DOCX) [file pone.0188211.s005.docx]

Results of EC25 bacteriological tests

Test Result

Gram staining Gram-negative, short rods

Motility in 0.35% agar Positive

Growth at 43^o^C 0.325% H_3_BO_3_ Positive

Hemolysis Gamma (non-hemolytic)

Gelatin liquefaction Negative

Indole production Positive

Catalase Positive

Oxidase Negative

β-galactosidase Positive

Lysine decarboxylase Positive

Urease Negative

DNAse Negative

Production of H_2_S Negative

Acetate utilization Positive

Citrate utilization Negative

Nitrate utilization Positive

D-arabinose fermentation* Positive

D-glucose fermentation* Positive

Fructose fermentation* Positive

Inositol fermentation* Negative

Lactose fermentation* Positive

D-mannitol fermentation* Positive

D-sorbitol fermentation* Positive

Sucrose fermentation* Positive

*On McConkey agar
